# Molecular Dissection Unveiling Dwarfing Effects of Plant Growth Retardants on Pomegranate

**DOI:** 10.3389/fpls.2022.866193

**Published:** 2022-03-10

**Authors:** Jingjing Qian, Ning Wang, Wenxu Ren, Rufan Zhang, Xiyao Hong, Lingyue Chen, Kaijing Zhang, Yingjie Shu, Nengbing Hu, Yuchen Yang

**Affiliations:** ^1^College of Agriculture, Anhui Science and Technology University, Fengyang, China; ^2^State Key Laboratory of Biocontrol, School of Ecology, Sun Yat-sen University, Guangzhou, China; ^3^State Key Laboratory of Biocontrol, School of Life Science, Sun Yat-sen University, Guangzhou, China; ^4^School of Materials Science and Engineering, Sun Yat-sen University, Guangzhou, China

**Keywords:** dwarfing, plant growth retardant, pomegranates, adverse impact, auxin biosynthesis

## Abstract

Dwarfed stature is a desired trait for modern orchard production systems. One effective strategy for dwarfing cultivation is exogenously applying plant growth retardants (PGRs) to plants. However, for many economic fruit trees, the current knowledge on the regulatory mechanisms underlying the dwarfing effect of PGRs is limited, which largely restricts the agricultural application of PGRs. In this study, we exogenously applied three kinds of PGRs [paclobutrazol, daminozide (B9), and mannitol] to the seedlings of pomegranate (*Punica granatum* L.) and performed comparative transcriptome analysis to elucidate the molecular features of PGR-induced dwarfing in pomegranates. Our results showed that all the three PGRs could significantly suppress plant growth of pomegranate. The inhibition of auxin biosynthetic processes, as well as auxin-mediated shoot development, may be considered as the main reason for the dwarfing. Besides that, different PGRs were also found to induce dwarfing *via* specific mechanisms, for example, cellular response to strigolactone was particularly suppressed by the application of paclobutrazol, while the level of carbohydrate homeostasis and metabolism were downregulated in conditions of either B9 or mannitol treatments. Furthermore, exogenous PGR application was supposed to cause adverse impacts on the normal physiological process of pomegranate seedlings, which may bring extra burden to pomegranate plants. These novel findings unveiled the genetic basis underlying the dwarfing in pomegranates, which provides deeper insights into PGR-mediated dwarfing cultivation of pomegranates.

## Introduction

For modern orchards, dwarfed stature is a favorable agronomic trait of economic fruit trees, which reduces the nutrients that plants spend on shoots and leaves and promotes flower blooming and fruit growing. In practical applications, dwarfing cultivation shows great advantages in improving the efficiency of land and light usages, increasing fruit yield and reducing the labor cost during the picking and spraying process, and has achieved some success (Knäbel et al., 2015; Foster et al., 2017; Wang et al., 2018; Cui et al., 2021; Zhou and Underhill, 2021). At present, there are several widely used strategies for plant dwarfing, such as breeding dwarf varieties (Patzak et al., 2012; Ma et al., 2014; Wang et al., 2018), grafting using certain rootstocks or interstocks (Sugar and Mielke, 2002; Foster et al., 2015, 2017), and applying exogenous plant growth regulators (Koukourikou-Petridou, 1996; Zhang et al., 2018, 2020; Shi et al., 2021). Unlike model species, it is more difficult to develop ideal dwarfing varieties or suitable rootstocks/interstocks for the fruits that are less investigated. Comparatively, exogenous application of growth regulators overcomes the shortcomings of lacking background knowledge and mature breeding system and shows apparent benefits in reducing the length and endeavors of cultivations.

Many plant growth regulators involved in cell division and elongation have shown their successes in dwarfing, such as brassinosteroid (BR), cytokinin, paclobutrazol, and daminozide (B9) (Koukourikou-Petridou, 1996; Zhang et al., 2018, 2020; Dong et al., 2019; Shi et al., 2021). Of them, paclobutrazol and B9 are also known as “plant growth retardants” (PGRs), and they repress plant growth and development *via* inhibiting the biosynthesis of gibberellins (GAs) and auxin [indole-3-acetic acid (IAA); hereafter we use auxin and IAA interchangeably] (Shi et al., 2021). In addition, exogenous application of mannitol, especially into the medium of *in vitro* tissue culture, has also been reported to restrict plant growth by provoking drought stimulus to plants (Bhat and Chandel, 1993). However, for non-model fruit species, the genetic mechanisms underlying PGR-induced dwarfing are still largely unknown, which restricts their agricultural applications. Furthermore, a better understanding of the genes and pathways related to PGR-induced dwarfing could provide clues for molecular-assisted screening and breeding of dwarfing varieties in the future.

Pomegranates (*Punica granatum* L.) is one kind of fruit crop widely cultivated throughout the Old World and the trees, in general, grow 5–7 m high. As a healthy fruit with high nutritional and medical values, the pomegranate industry has sprung up in the global consumer market in recent years (Bourekoua et al., 2018; Shahamirian et al., 2019; Asrey et al., 2020; Turrini et al., 2020). Like other fruit trees, dwarfing the tree size of pomegranates is supposed to reduce management costs and increase profits. However, due to the lack of studies on pomegranates, our knowledge of the molecular mechanisms underlying dwarfing in pomegranates is still limited.

In the current study, we first examined the dwarfing effects of exogenous PGRs, paclobutrazol, B9, and mannitol, on pomegranate seedlings during *in vitro* tissue culture. We then performed comparative transcriptome analysis to explore the genetic basis underlying the dwarfing effects of each PGR, which may provide new insights into dwarfing cultivation of pomegranates.

## Materials and Methods

### Plant Materials and Plant Growth Retardant Treatment

All the plant materials used in this study were collected from the “Hongmanao” pomegranates planting base of Anhui Science and Technology University. Tips of 2–3 cm were retrieved from the tender stems of the pomegranates well grown and free of diseases and insect pests, and cultivated in water for 2–3 days. Then, the growth point of each stem tip was collected and rinsed with clean water in a beaker for 15 min, 75% alcohol for 30 s, and sterile water 2–3 times, to remove surface dust and other impurities. Rinsed materials were then disinfected with 0.1% HgCl_2_ for 8 min and rinsed with sterile water 3–5 times. The growth point of the leaf primordium (approximately 0.3–0.5 mm) was collected from the disinfected materials and inoculated to a prepared culture medium. The explants were grown in the WPM medium (containing 0.8 mg/L IBA 3%, sucrose, and 0.55% agar under a pH of 6.5) at the culture chamber under controlled culture conditions: a 12/12 h light/dark photoperiod of 3,000 LX illumination, 25 ± 1°C temperature and 50% relative humidity.

After three cycles of subculture (35–40 days for each cycle), 0.8 mg/L IBA was added to the WPM culture media to induce the proliferation of stem tips. Then, the seedlings of the same growth condition were selected and assigned into four experimental groups, where the first three groups were treated with paclobutrazol, B9, and mannitol, respectively, and the remaining one group was treated with the same amount of distilled water as the control (CK). As shown in Supplementary Table 1, five concentration gradients were set for each drug, and each treatment/concentration included five plants as biological replicate. After 30, 60, and 90 days of treatment, we measured the height of the above-ground parts of each plant in each treatment. According to their growth conditions, plants treated at two desired concentrations were selected for each PGR (6 and 8 mg/L for paclobutrazol, 6 and 8 mg/L for B9, and 2.5 and 15 g/L for mannitol) for downstream experiments. Leaves were collected from each selected plant with the treatment of 90 days and activities of antioxidant enzymes, including superoxidase dismutase (SOD) and peroxidase (POD), and the reduced glutathione/oxidized glutathione (GSH/GSSG) ratio were measured following the methods described in Qian et al. (2020) to assess the physiological status of plants under treatment. Meanwhile, fresh leaves were also collected from two plants (biological replicates) of each treatment of 90 days (CK and PGRs), frozen in liquid nitrogen and stored at −80°C for RNA sequencing (RNA-seq).

### RNA Sequencing and Differential Expression Analysis

Total RNA extraction, RNA-seq, and data preprocessing were carried out as described in Qian et al. (2020). High-quality reads were aligned to the *P. granatum* reference genome (Luo et al., 2020) using hisat2 program (Kim et al., 2019). The reads mapped to more than one genome location were filtered out and only those uniquely mapped were retained for downstream analyses. For each gene, the expression level was assessed by the abundance of mapped reads using the featureCounts program from the Subread package (Liao et al., 2014). DESeq2 (Love et al., 2014) was employed for differential expression analysis between each of the PGR treatments (paclobutrazol, B9, and mannitol of different concentrations) and CK. Genes whose |log_2_ fold-change _*treatment/CK*_| > 1 and adjusted *p* < 0.05 were considered as significantly differentially expressed (denoted as “DEGs”). Hereafter, DEGs are denoted as “upregulated” or “downregulated” if their expression after treatment was higher or lower than CK. Raw read counts of genes were normalized by the fragments per kilobase of exon per million fragments mapped (FPKM), to correct between-sample differences in sequencing depth.

### Functional Enrichment and Trend Analysis

Genome-wide functional annotations, including Gene Ontology (GO) and Kyoto Encyclopedia of Genes and Genomes (KEGG) annotations, were adopted from our previous study (Qian et al., 2020). For each of the comparisons between PGR-treated plants and CK, GO and KEGG enrichment analyses were implemented for up- and downregulated DEGs, respectively. The significance levels were assessed by Fisher’s exact test, where GO terms and KEGG pathways were considered as significantly overrepresented if their *p* < 0.05.

To access the dose-effect of each PGR (paclobutrazol, B9, and mannitol), trend analysis was carried out across different treatment concentrations using Mfuzz package (Futschik and Carlisle, 2005; Kumar and Futschik, 2007). Briefly, for each treatment, highly variable genes were grouped in six clusters *via* mfuzz function, according to the transcriptional similarities across different treatments. Then, the significantly enriched clusters were identified by Fisher’s exact test as described in Qian et al. (2020).

## Results

### Plant Height and Antioxidative Enzyme Activities Under the Treatment of Plant Growth Retardants

**FIGURE 1:**
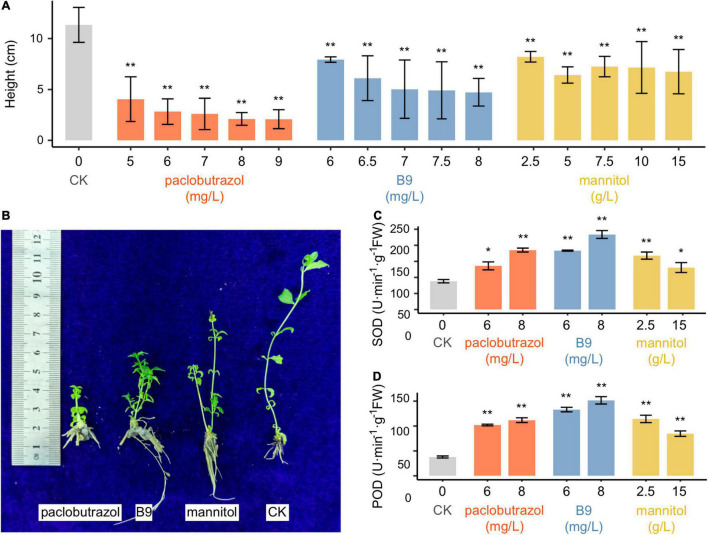
Plant height and antioxidative enzyme activities of pomegranate seedlings treated with paclobutrazol, B9, and mannitol for 90 days. **(A)** Height of seedlings of CK (untreated) or treated with three PGRs of different concentrations; **(B)** an image showing CK and treated seedlings; **(C)** SOD activity; **(D)** POD activity. Values are mean ± SD. *p < 0.05; **p < 0.01.

Compared to untreated conditions (CK), all the PGRs were found to significantly suppress the growth of pomegranate seedlings (Figures 1A,B and Supplementary Figure 1). After 30 days of treatments, plants treated with paclobutrazol showed the most reduction in height (reduced by 74.0–79.4% compared to CK; 1.2–1.6 cm in average for five concentrations vs 6.0 cm for CK; *p* < 0.01 for all the five concentrations), followed by mannitol and B9 (Supplementary Figure 1A). When extended to 60 days, the height of the paclobutrazol-treated plants was only 13.2–18.0% of that of CK (*p* < 0.01), and the plant height under the treatment of mannitol was slightly higher than that under B9 treatment (Supplementary Figure 1B). After 90 days, the height ranged from 2.1 to 4.1 cm with the paclobutrazol treatment from 5 to 9 mg/L, which were 64.2–81.6% shorter than that of CK (11.3 cm in average) (*p* < 0.01; Figure 1A). Comparatively, the average height of B9- and mannitol-treated seedlings was 4.7–7.9 and 6.4–8.2 cm, respectively, which were also significantly shorter than CK (*p* < 0.01; Figure 1A).

Under the treatments of both paclobutrazol and B9, the activities of SOD and POD, as well as the GSH/GSSG ratio, in seedlings were found to be substantially enhanced for each of the PGR treatments than CK (*p* < 0.05 or <0.01 for all comparisons between treated plants and CK; Figures 1C,D and Supplementary Figure 2). For both paclobutrazol and B9, the enzymes were more activated as the increasing of treatment concentrations that SOD and POD activity increased by 169.9–301.0 and 54.1–164.8% over CK, respectively. In contrast, the activities were lower with the mannitol treatment of high concentration (15 g/L) than the low concentration (2.5 g/L). It might be due to the poor growth condition when treated with over-dose mannitol. These results suggested that, besides dwarfing, these exogenous PGR supplies may also lead to adverse impacts on plant normal physiological status.

### Transcriptomic Profiling in Conditions of Plant Growth Retardant Treatments

**FIGURE 2:**
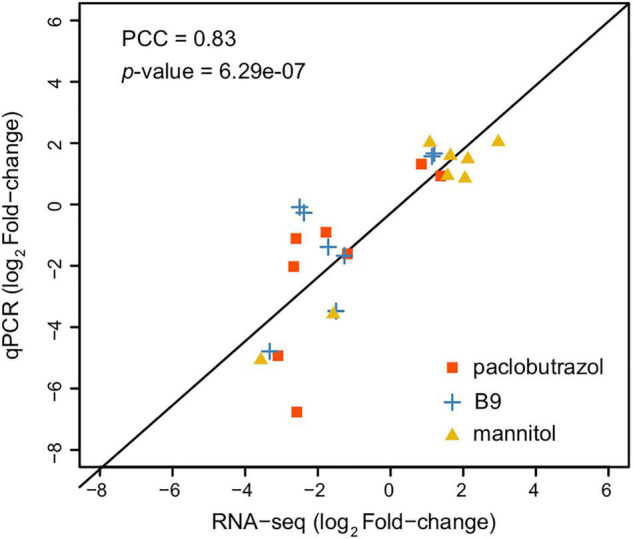
Correlation between the log-scaled expression level changes (treatment group vs CK) inferred from RNA-seq (*x*-axis) and of qPCR (*y*-axis). Data from different treatment groups (paclobutrazol, B9, and mannitol) are highlighted in different colors. Pearson correlation coefficient (PCC) and *p* are shown in the plot.

In a total, 23.87–30.76 million raw reads were obtained from RNA-seq, where 23.81–30.61 million reads passed the quality control. After alignment, 747 and 3,917 DEGs were detected under 6 and 8 mg/L treatments of paclobutrazol, respectively (Supplementary Figures 3A,B), and 3,132 and 2,793 genes were differentially expressed when treated with 6 and 8 mg/L B9 (Supplementary Figures 3C,D). We further identified 1,132 and 815 DEGs under the treatments of 2.5 and 15 g/L mannitol (Supplementary Figures 3E,F). The results of qPCR validation showed a high correlation between the gene expression levels from RNA-seq and qPCR (Pearson correlation coefficient = 0.83; *p* = 6.29e-7; Figure 2), indicating a reliable inference of gene expression by our RNA-seq data.

### Functional Enrichment and Trend Analysis Unveiling Transcriptional Responses to Plant Growth Retardant Treatments

**FIGURE 3:**
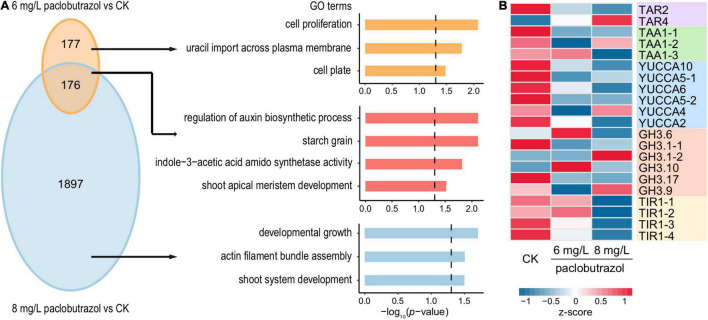
Overview for the downregulated DEGs in pomegranates treated with paclobutrazol. **(A)** Overlap and functional enrichment analysis of DEGs downregulated under 6 mg/L paclobutrazol over 8 mg/L paclobutrazol. **(B)** Heatmap showing gene expression profile of the downregulated DEGs related to IAA biosynthetic and signaling pathways in conditions of CK, and 6 and 8 mg/L paclobutrazol treatments.

For paclobutrazol, after the treatment of 6 mg/L, there were 394 up- and 353 downregulated DEGs, respectively (Supplementary Figure 3A). Comparatively, 1,844 and 2,073 genes were over- and underexpressed when treated with 8 mg/L (Supplementary Figure 3B). For downregulated DEGs, 176 were shared between the two treatment concentrations. GO enrichment analysis showed that these commonly suppressed genes were mainly involved in shoot apical meristem development and auxin biosynthesis and metabolism (Figure 3A). Comparatively, the genes specifically inhibited by 6 mg/L treatment were enriched for the cell proliferation process, while those specific to 8 mg/L treatment were relevant to developmental growth, actin filament bundle assembly, and shoot system development (Figure 3A). We then investigated the expression levels of three key genes along the tryptophan-dependent IAA biosynthetic pathway, including those encoding tryptophan aminotransferase-related protein (TAR), L-tryptophan–pyruvate aminotransferase 1 (TAA1), and indole-3-pyruvate monooxygenase (YUCCA) (Mashiguchi et al., 2011; Mano and Nemoto, 2012). The results showed that, in general, the expression of these genes was suppressed by paclobutrazol treatments, at both concentrations (Figure 3B). In particular, most of the *TAA1* and *YUCCA* genes were downregulated under the treatment of 8 mg/L paclobutrazol. Comparatively, there was no consistent pattern across different members of indole-3-acetic acid-amido synthetase (GH3) genes, which respond to an excess amount of auxin in plants (Mashiguchi et al., 2011).

Trend analysis showed different impacts across the treatments of different paclobutrazol doses. One of the two sufficiently overrepresented trends was the genes were of similar level of downregulation under the treatment of either 6 or 8 mg/L, which were mainly involved in cell cycle, DNA replication proteins and shoot apical meristem development (Supplementary Figure 4A). For the second scenario, the genes were of more reduced expression as the increase of paclobutrazol concentration, and these genes were found to participate in growth hormone synthesis and primary shoot apical meristem specification (Supplementary Figure 4B), indicating the plausible mechanism underlying the severer growth suppression induced by the increase of paclobutrazol dosage (from 6 to 8 mg/L). Meanwhile, for the treatments of both concentrations, genes responding to oxidative stress were observed to be upregulated (Supplementary Figures 4C,D), which was corresponding to the enhanced activities of antioxidant enzymes (Figures 1B,C). It may reflect a stress response in paclobutrazol seedlings under the treatment of paclobutrazol.

Concerning B9, 1,726 and 1,410 genes were significantly downregulated after 6 and 8 mg/L treatment (Supplementary Figures 3C,D, 5A). For both two treatments, genes involved in BR metabolism, chromocenter, and actin filament bundle assembly were suppressed compared to CK (Supplementary Figure 5B). Each treatment also has specific impacts on pomegranates: auxin biosynthetic regulation and cell wall modification during cell growth were downregulated under 6 mg/L B9 treatment (Supplementary Figure 5C), while cell division, developmental growth, and ABA degradation processes were exclusively depressed when treated with 8 mg/L B9 (Supplementary Figure 5D). Comparatively, genes related to oxidative stress resistance were significantly induced by the treatments (Supplementary Figures 5E–H). Trend analysis showed that plant growth and primary shoot apical meristem specification were more suppressed with the increase of concentration (from 6 to 8 mg/L), while the antioxidative processes, such as hydrogen peroxide response and peroxisome and glutathione metabolism, became stronger accordingly (Supplementary Figure 6).

**FIGURE 4:**
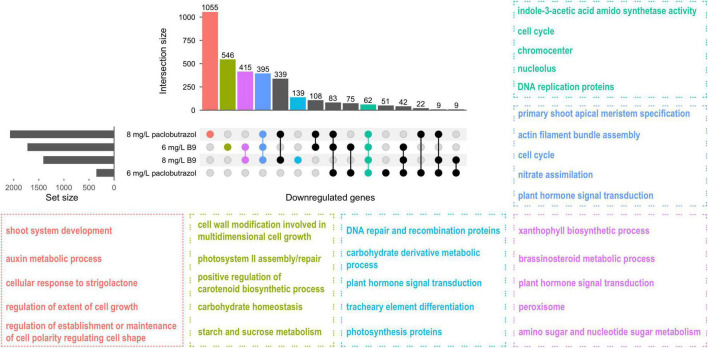
Overlap and functional enrichment analysis of DEGs downregulated under the treatments of paclobutrazol and B9. Different colors represent feature-enriched GO terms and KEGG pathways for different categories of downregulated DEGs.

We also assessed the different transcriptional responses between paclobutrazol- and B9-treated plants. In total, 62 genes were downregulated under all four treatments, and they mainly participated in regulating IAA synthetase and cell cycle, including the processes of DNA replication and chromocenter (Figure 4). Moreover, 395 genes were commonly suppressed when treated with 8 mg/L paclobutrazol and 6 and 8 mg/L B9, which were enriched for shoot apical meristem specification, cell cycle regulation, and plant hormone signal transduction (Figure 4). It is noteworthy that, besides these commonly depressed pathways, the processes involved in strigolactone response, shoot system development, and auxin metabolism were inhibited exclusively by 8 mg/L paclobutrazol, while carbohydrate-derivative metabolic process was suppressed specifically by 8 mg/L B9 treatment (Figure 4), suggesting different regulatory mechanisms underlying the dwarfing by different PGRs.

**FIGURE 5:**
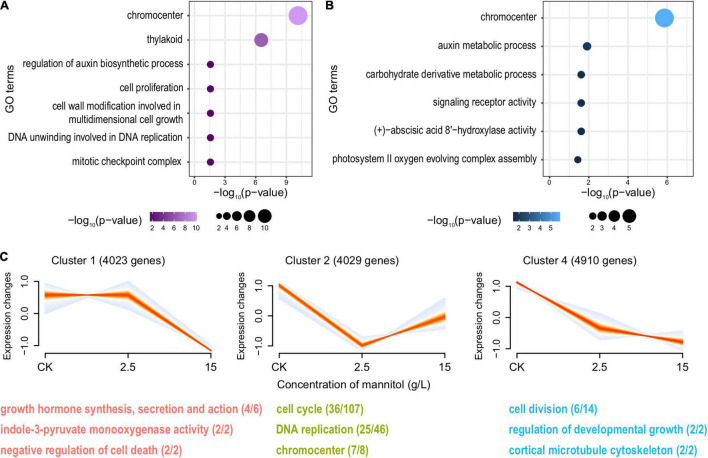
Overview for the downregulated DEGs in pomegranates treated with mannitol. Functional enrichment analysis for downregulated DEGs in pomegranates under the treatment of 2.5 g/L **(A)** and 15 g/L mannitol **(B)**. **(C)** Significant clusters of genes with a similar trend in expression across different mannitol concentrations (0, 2.5, and 15 g/L) and the representative GO terms and KEGG pathways enriched for each set of genes. For each panel, each line represents the expression change of one gene across different concentrations, and the intensity of colors represents the membership value of genes (the more red, the greater membership values; the more blue, the lesser membership values). The number of genes belonging to each cluster is shown in the parentheses after the cluster label. For each GO term or KEGG pathway, the number of genes is shown in parentheses, with the number before and after the forward-slash representing the genes exhibiting the trend and the genes from the genome background.

When treated with mannitol, 451 up- and 681 downregulated genes were induced at 2.5 g/L (Supplementary Figure 3E), while the expression level of 381 and 434 genes was increased or reduced at 15 g/L (Supplementary Figure 3F). For both treatments, the auxin biosynthetic process was significantly suppressed (Figures 5A,B). Corresponding to our expectation, the expression of genes related to cell division and plant developmental growth was even reduced when treated with 15 g/L compared to 2.5 g/L. In contrast, cell proliferation and DNA replication processes were more inhibited under the treatment of 2.5 g/L than 15 g/L (Figure 5C). It is noteworthy that, the pathways involved in the response to oxidative stress, such as the removal of reactive oxygen species (ROS) and the regulation of osmotic equilibrium, were largely enhanced (Supplementary Figure 7), suggesting the undesired side effect of mannitol on plants. Furthermore, this hazardous impact got even worse with the increase of treatment concentrations. Compared to 2.5 g/L treatment, genes of the negative regulation of cell death showed reduced expressions when treated with 15 g/L mannitol (Supplementary Figure 7).

## Discussion

Plant growth retardants have been widely applied for crop and fruit dwarfing (Koukourikou-Petridou, 1996; Zhang et al., 2018, 2020; Shi et al., 2021). Because of the specific characteristics of each chemical, different PGRs are supposed to perform differently on plant growth, in terms of both morphological and physiological properties (Dong et al., 2019; Shi et al., 2021). A comprehensive investigation of the dwarfing effects on economic fruits would largely broaden their agricultural applications.

In this study, we assessed the impacts of three typical PGRs, paclobutrazol, B9, and mannitol, on pomegranate dwarfing, and found that all three resulted in a significant reduction of growth in pomegranate seedlings. In particular, paclobutrazol treatment caused notably less growth despite being applied at low concertation (e.g., 5 mg/L), which was consistent with previous reports (Porlingis and Voyiatzis, 1985; Jaini et al., 2014; Shi et al., 2021). Transcriptome analysis showed that IAA biosynthesis was inhibited by the treatments of all the three PGRs (Figures 3, 5A,B). As an important plant hormone, IAA has essential roles in regulating plant growth and development (Davies, 2010). Compared to untreated plants, expression of the key genes for IAA biosynthesis and metabolism were decreased by either 6 or 8 mg/L paclobutrazol treatments (Figure 3B). Of them, *TAA1*, *TAR2*, and *YUCCA* genes catalyze the conversion of tryptophan (Trp) to indole-3-pyruvic acid (IPA) and IPA to IAA along tryptophan-independent IAA biosynthetic pathway (Mashiguchi et al., 2011; Mano and Nemoto, 2012); hence, silencing of these genes caused substantial endogenous auxin deficiency in pomegranate seedlings. In addition, genes encoding TRANSPORT INHIBITOR RESPONSE (TIR1) were also depressed by paclobutrazol treatments (Figure 3B), which reduced inhibition on the degradation of Aux/IAA transcriptional repressors (Villalobos et al., 2012) and thus downregulated IAA-regulated transcription. The suppression of IAA-related processes would further trigger subsequent downregulation of cell proliferation and shoot apical meristem development (Figure 3A), finally leading to the reduced shoot growth of pomegranate seedlings. These results highlighted the core role of PGR-induced IAA depletion in pomegranate dwarfing.

Besides the common auxin-suppressing pathway, different PGRs were also revealed to dwarf plants *via* different mechanisms. Compared to B9, 8 mg/L paclobutrazol treatment specifically inhibited cellular response to strigolactone (Figure 4). In Arabidopsis, defects in strigolactone synthesis led to a substantial reduction in plant height (Bennett et al., 2016). Hayward et al. (2009) reported a dynamic feedback loop of auxin and strigolactone in regulating shoot development. Specifically, a decrease in auxin level would relax the inhibition of IAA12 on *MAX3-* and *MAX4*-mediated strigolactone production, resulting in depleted strigolactone, while strigolactone deficiency blocks the export of auxin from buds into the polar auxin transport and reduces auxin gradients throughout plant body, which are required for plant growth and development (Hayward et al., 2009; Zhao, 2010). It suggested that paclobutrazol-mediated dwarfing involves the synergies of multiple plant hormones. Comparatively, carbohydrate homeostasis and metabolism, as well as cell wall modification, were specifically downregulated by the treatments of B9 or mannitol (Figures 4, 5A,B). These results mirrored that different PGRs induced plant dwarfing *via* different regulatory mechanisms.

Despite the positive role in dwarfing, we observed increased activities of antioxidative enzymes in pomegranate seedlings under the treatment of exogenous PGRs. Compared to CK, PGR treatments significantly increased the activities of antioxidant enzymes in pomegranate seedlings (Figures 1B,C and Supplementary Figure 2). Consistently, the genes involved in redox state responding and superoxide radical removal were overexpressed under PGR treatments (Supplementary Figures 4C,D, 5F, 7). In addition, the level of GSH, as well as the corresponding GSH synthesis and metabolism, were also apparently induced in the condition of PGR treatments (Supplementary Figures 4C,D, 5H, 7). Such an enhancement was also observed in other PGR-mediated dwarfed plants, such as *Magnolia wufengensis* and *Chlorophytum capense*, and it was previously considered as a sign of improved stress resistance in plants (Zhou, 2017; Shi et al., 2021). However, as important antioxidative systems in plant cells, POD, SOD, and GSH are generally induced to scavenge the accumulation of endogenous harmful substances, such as ROS, under stress conditions (Mittler, 2002; Belmonte et al., 2005; Hellou et al., 2012). Thus, their increased activities in the presence of PGRs are more likely to be a byproduct of the adverse impacts elicited by PGRs on the normal physiological processes of pomegranate seedlings, rather than a signature of the direct promotion of stress tolerance capacity. In addition, for paclobutrazol and B9, SOD, and POD activities became higher as the treatment dosage increased, although dwarfing influence on shoot growth became slightly stronger as well (Figures 1B,C and Supplementary Figures 6A,C). It suggested stronger antioxidative responses being elicited to mitigate the higher degree of PGR-provoked oxidative damage, and thus promote the tolerance in pomegranates to the adverse effects of PGRs. Mannitol is the only exception that POD and SOD activities decreased as the concentration increased from 2.5 to 15 g/L (Figures 1B,C), which may be attributed to the worse growth condition induced by excess amount of mannitol (personal observation). This is further supported by the upregulation of cell death when treated with 15 g/L mannitol (Figure 5C). It is noteworthy that PGR-induced transcriptional responses may cause further influences, either positive or negative, on the plant tolerance to other external stresses. It may pose challenges to agricultural production of pomegranates, especially under the current drastic fluctuation of global climate. However, our current knowledge on the burden of exogenous PGR application is still limited. Further investigations are required to assess the optimal PGR dosage that could balance desired dwarfing effects and undesired reduction in plant physiological status and environmental adaptation.

## Conclusion

In the current study, we dissected the molecular mechanisms underlying PGR-induced dwarfing in pomegranates. Dwarfing induced by all the three kinds of PGRs involves the inhibition of auxin biosynthetic and metabolic processes and IAA-mediated shoot development. Different genes and pathways were found to be suppressed by different PGR treatments, reflecting the specificity of different PGRs in eliciting plant dwarfing. Furthermore, we showed that exogenous PGR application causes adverse impacts on the normal physiological process of pomegranate seedlings, which may bring extra burden to pomegranate plants. These novel findings could better assist the application of PGRs in dwarfing cultivation of pomegranates, as well as the molecular-aided screening and breeding of dwarfed pomegranate varieties in the future.

## Data Availability Statement

The datasets presented in this study can be found in online repositories. The name of the repository and accession number can be found below: National Center for Biotechnology Information (NCBI) Gene Expression Omnibus (GEO), https://www.ncbi.nlm.nih.gov/geo/, GSE195722.

## Author Contributions

JQ and YY designed the study. NW, JQ, KZ, YS, and NH collected the materials and performed the experiments. WR, RZ, XH, LC, and YY analyzed and interpreted the data. NW, WR, JQ, and YY wrote the manuscript. All authors contributed to the revision and approved the final manuscript.

## Conflict of Interest

The authors declare that the research was conducted in the absence of any commercial or financial relationships that could be construed as a potential conflict of interest.

## Publisher’s Note

All claims expressed in this article are solely those of the authors and do not necessarily represent those of their affiliated organizations, or those of the publisher, the editors and the reviewers. Any product that may be evaluated in this article, or claim that may be made by its manufacturer, is not guaranteed or endorsed by the publisher.
