# Supplementary material for: Molecular Dissection Unveiling Dwarfing Effects of Plant Growth Retardants on Pomegranate
Source: Front Plant Sci. 2022 Mar 10;13:866193. doi: 10.3389/fpls.2022.866193 (PMC8961278; doi:10.3389/fpls.2022.866193)
Supplement: Supplementary file 1 [file Data_Sheet_1.docx]

**Supplementary materials for Online Publication**

**Molecular dissection unveiling dwarfing effects of plant growth retardants on pomegranate**

Jingjing Qian^1†^, Ning Wang^1†^, Wenxu Ren^2†^, Rufan Zhang^3^, Xiyao Hong^4^, Lingyue Chen^2^, Kaijing Zhang^1^, Yingjie Shu^1^, Nengbing Hu^1^, Yuchen Yang^2*^

1. Anhui Science and Technology University, Fengyang, Anhui 233100, China

2. State Key Laboratory of Biocontrol, School of Ecology, Sun Yat-sen University, Guangzhou 510275, China

3. State Key Laboratory of Biocontrol, School of Life Science, Sun Yat-sen University, Guangzhou 510275, China

4. School of Materials Science & Engineering, Sun Yat-sen University, Guangzhou 510006, China.

^†^ These authors contributed equally to this work.

^*^ Corresponding author

**Table S1** PGR concentration for each treatment

|  | Treatment group | | | | |
| --- | --- | --- | --- | --- | --- |
|  | 1 | 2 | 3 | 4 | 5 |
| paclobutrazol (mg/L) | 5 | 6 | 7 | 8 | 9 |
| B9 (mg/L) | 6 | 6.5 | 7 | 7.5 | 8 |
| mannitol (g/L) | 2.5 | 5 | 7.5 | 10 | 15 |


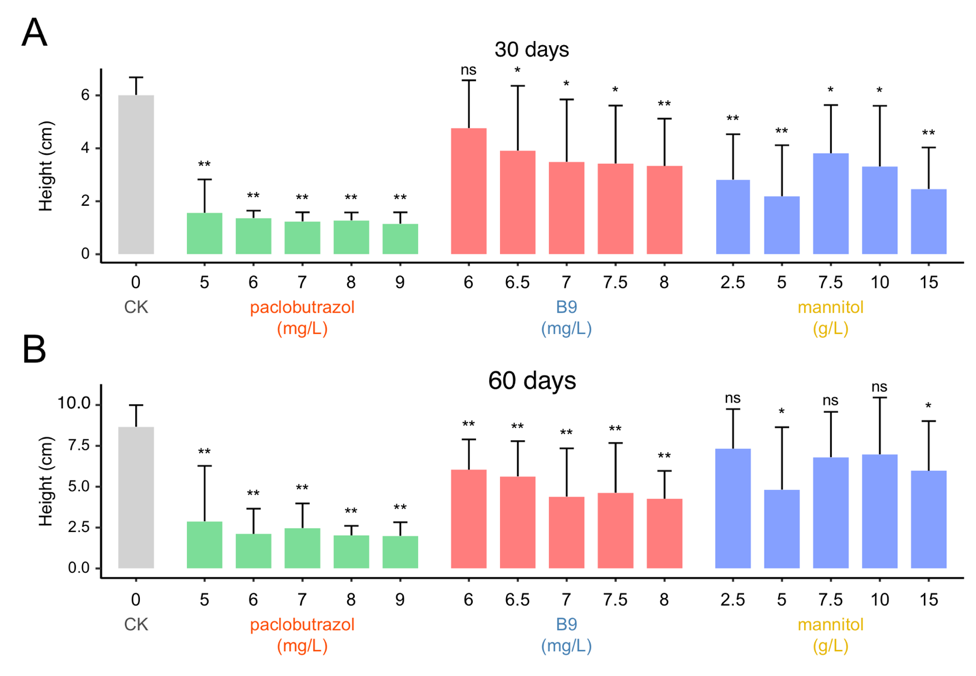


**Figure S1.** Plant height of pomegranate seedlings of CK (untreated) or treated with three PGRs of different concentrations for 30 (**A**) and 60 days (**B**). Values are mean ± SD. ns, nonsignificant; *, *p*-value < 0.05; **, *p*-value < 0.01.

**
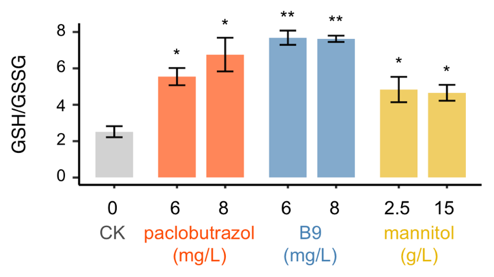
**

**Figure S2.** GSH/GSSG ratio in pomegranate seedlings of CK (untreated) or treated with three PGRs of different concentrations for 90 days. Values are mean +/- SD. *, *p*-value < 0.05; **, *p*-value < 0.01.

**
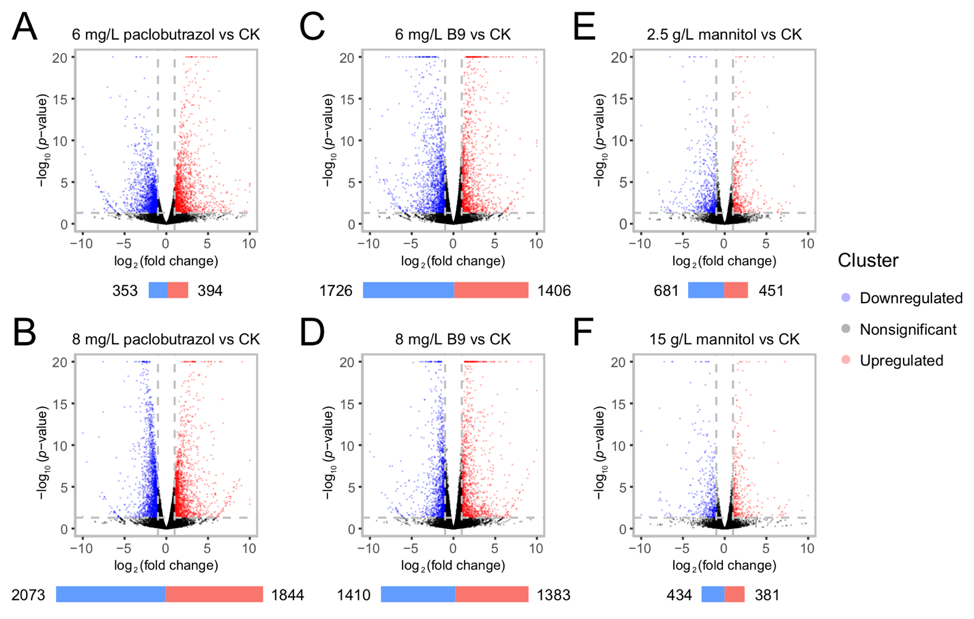
**

**Figure S3**. Volcano plots for differentially expressed genes (DEGs) in pomegranate seedlings treated with paclobutrazol, B9 and mannitol of different concentrations compared to CK (**A**: 6 mg/L paclobutrazol vs CK; **B**: 8 mg/L paclobutrazol vs CK; **C**: 6 mg/L B9 vs CK; **D**: 8 mg/L B9 vs CK; **E**: 2.5 g/L mannitol vs CK; **F**: 15 g/L mannitol vs CK). Different colors represent different clusters of genes (blue: downregulated DEGs; red: upregulated DEGs; grey: nonsignificant genes).

**
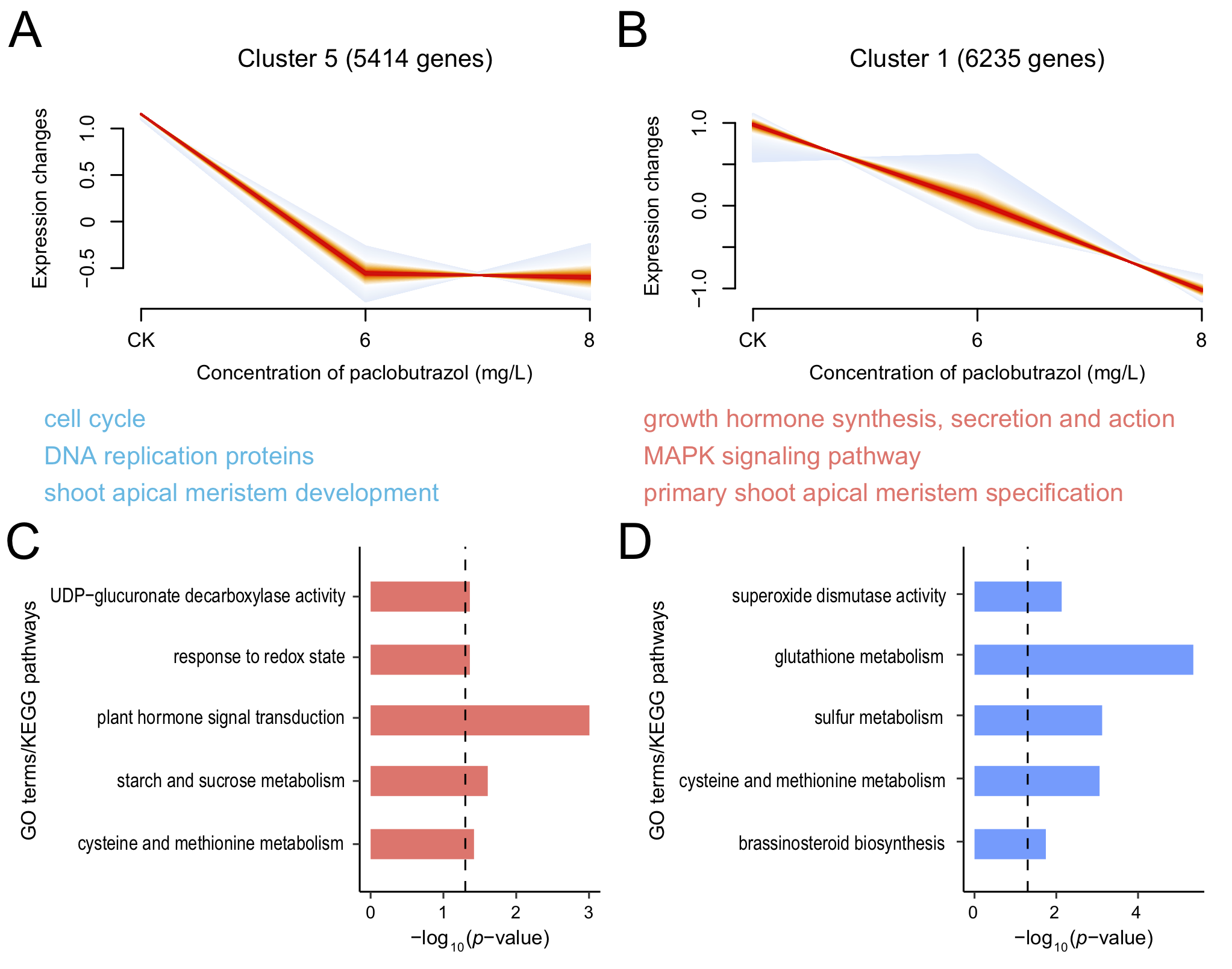
**

**Figure S4.** **A-B.** Significant clusters of genes with similar trend in expression across different paclobutrazol concentrations (0, 6 and 8 mg/L) and the representative GO terms and KEGG pathways enriched for each set of genes. For each panel, each line represents the expression change of one gene across different concentrations and the intensity of colors represent the membership value of genes (the more red, the greater membership values; the more blue, the less membership values). The number of genes belonging to each cluster is shown in the parentheses after the cluster label. **C-D.** Functional enrichment analysis for upregulated DEGs in pomegranates under the treatment of 6 mg/L (**C**) and 8 mg/L paclobutrazol (**D**).


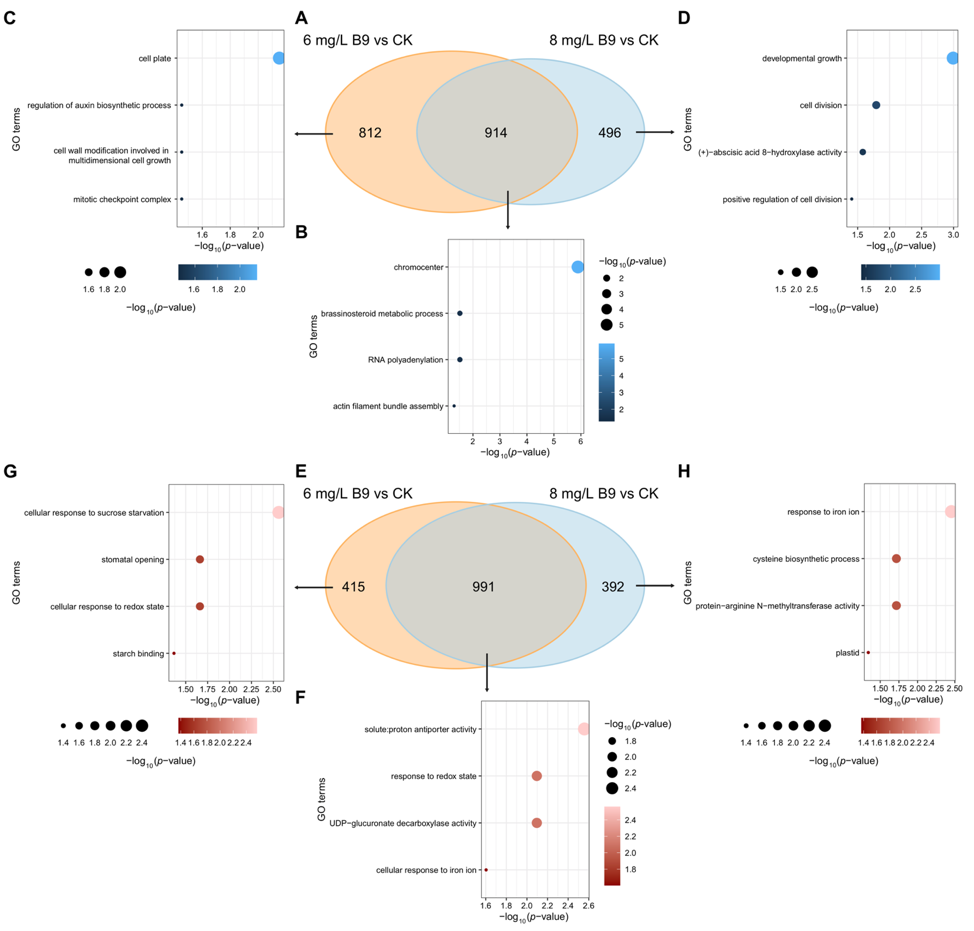


**Figure S5.** Overlap and functional enrichment analysis of DEGs downregulated (**A-D**) and upregulated (**E-H**) under the treatments of 6 and 8 mg/L B9.


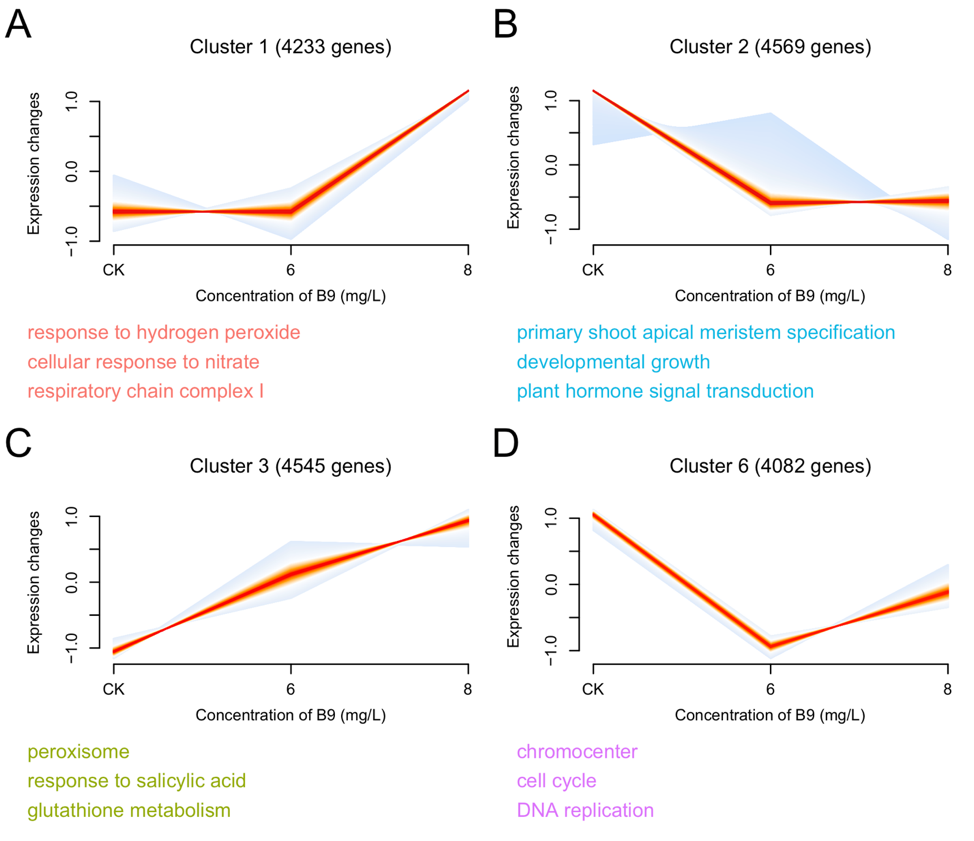


**Figure S6.** Significant clusters of genes with similar trend in expression across different B9 concentrations (0, 6 and 8 mg/L) and the representative GO terms and KEGG pathways enriched for each set of genes. For each panel, each line represents the expression change of one gene across different concentrations and the intensity of colors represent the membership value of genes (the more red, the greater membership values; the more blue, the less membership values). The number of genes belonging to each cluster is shown in the parentheses after the cluster label.


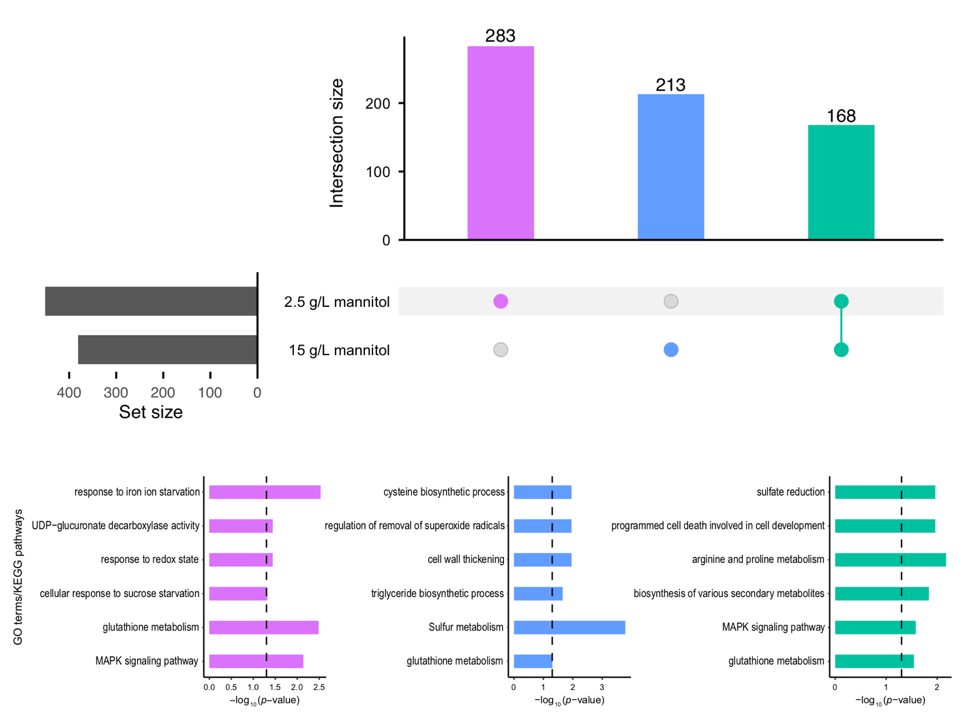


**Figure S7.** Overlap and functional enrichment analysis of DEGs upregulated under the treatments of 2.5 and 15 g/L mannitol. Different colors represent feature enriched GO terms and KEGG pathways for different categories of upregulated DEGs.
